# Competition (‘Steal’ Phenomenon) between [^68^Ga]Ga-PSMA-11 Uptake in Prostate Tumor Tissue Versus Healthy Tissue

**DOI:** 10.3390/pharmaceutics13050699

**Published:** 2021-05-11

**Authors:** Esmée C. A. van der Sar, Bart de Keizer, Marnix G. E. H. Lam, Arthur J. A. T. Braat

**Affiliations:** Department of Radiology and Nuclear Medicine, University Medical Center Utrecht, 3584 CX Utrecht, The Netherlands; B.deKeizer@umcutrecht.nl (B.d.K.); M.Lam@umcutrecht.nl (M.G.E.H.L.); A.J.A.T.Braat@umcutrecht.nl (A.J.A.T.B.)

**Keywords:** prostate cancer, PSMA, Gallium-68, uptake, steal phenomenon

## Abstract

We aimed to clarify whether a steal ‘phenomenon’ exists by investigating if uptake of ‘prostate specific membrane antigen’ (PSMA) in prostate tumor tissue correlates with the uptake in healthy tissue. Patients with prostate cancer referred for a [^68^Ga]Ga-PSMA-11 PET/CT were identified retrospectively. Semi-automated quantitative image analysis was performed; fractional healthy tissue [^68^Ga]Ga-PSMA-11 uptake volume (HT-PSMA (SUV*cm^3^)) in the lacrimal, submandibular, and parotid glands, and kidneys, and the fractional total lesion [^68^Ga]Ga-PSMA-11 uptake volume (TL-PSMA (SUV*cm^3^)) of prostate cancer were used. Ninety-two patients, age 78 ± 8 years, were analyzed. Median TL-PSMA was 703.37 SUV*cm^3^ (IQR 119.56–2778.20), median HT-PSMA of the lacrimal, submandibular, and parotid glands, and kidneys was: 13.69 (IQR 7.29–19.06), 194.75 (IQR 133.67–276.53), 552.54 (IQR 379.98–737.16), and 8092.75 SUV*cm^3^ (IQR 5793.02–11,385.86), respectively. A significant (*p*-value ≤ 0.001) but weak–moderate correlation was found between the TL-PSMA and HT-PSMA of the parotid- and submandibular glands, and kidneys (correlation coefficient of −0.447, −0.345, and −0.394, respectively). No correlation was found between TL-PSMA and HT-PSMA of the lacrimal glands. The existence of a ‘steal’ phenomenon cannot be confirmed in this study. Healthy tissue uptake of [^68^Ga]Ga-PSMA-11 is only partially influenced by TL-PSMA. Thus, modification of therapeutic PSMA activity should not be adjusted based on TL-PSMA alone.

## 1. Introduction

Prostate cancer is the second most common cancer in men in the Western world [1,2]. For men with metastatic prostate cancer the standard treatment options are limited to hormone therapy and chemotherapy. However, these therapies are associated with considerable side effects. For some patients, chemotherapy is also contraindicated or not well tolerated and, of the prostate cancer patients, a proportion of 10–20% will eventually progress to metastatic castration-resistant prostate cancer (CRPC) [3]. Therefore, a new therapy with less side effects for metastatic prostate cancer is desirable.

With the development of the small molecule called “prostate specific membrane antigen” (PSMA), a new diagnostic and therapeutic ligand for metastatic prostate cancer has become available. PSMA is a type II membrane glycoprotein, also known as folate hydrolase or glutamate carboxypeptidase II. It is highly overexpressed by the cell surface of 90–100% of all prostate cancers and has a 1000-fold lower expression in normal tissue [4,5,6]. The expression of PSMA increases with tumor aggressiveness, metastasis development, and disease recurrence [7,8]. Therefore, it is a useful tool for the imaging of metastatic prostate cancer using PSMA PET/CT, but also for the treatment using PSMA-targeted radioligand therapy [9,10]. However, there are some side effects documented with PSMA-targeted radioligand therapy; most commonly xerostomia (47–87%) in which several patients required salivary substitute gel, and in the case of hematotoxicity, mainly thrombocytopenia in 17–40% of the patients treated with [^177^Lu]Lu-PSMA-617 therapy [9,11]. An explanation can be found in the biodistribution of PSMA-tracer as there is also physiological uptake (and therefore irradiation) in healthy tissue such as the kidneys, liver, salivary glands, intestine, and to a lesser degree in the lacrimal glands, spleen, and prostate [9,12,13]. Some have suggested that competition between the uptake of the radiopharmaceutical in prostate tumor tissue and healthy tissue may influence the biodistribution by inducing a ‘steal’ phenomenon, resulting in diminished uptake in healthy tissue [14,15,16].

In the current study, we aimed to clarify whether a steal ‘phenomenon’ exists by investigating if PSMA uptake in tumor tissue correlates with the uptake in healthy tissue. If a ‘steal’ phenomenon indeed exists, PSMA-targeted radioligand therapy may be individualized based on the total tumor load and potentially reduce/avoid unwanted side effects.

## 2. Materials and Methods

### 2.1. Study Population

Patients referred for a [^68^Ga]Ga-PSMA-11 PET/CT were identified retrospectively from a single center from March 2016 to January 2019. Patients were imaged for either primary staging of prostate cancer, biochemical recurrence, (biochemical) progression, or evaluation prior to [^177^Lu]Lu-PSMA-617 therapy or [^223^Ra]Ra-dichloride therapy. Patients with recent serum prostate specific antigen (PSA) value (±1 month) and three or more metastases on [^68^Ga]Ga-PSMA-11 PET/CT were included. [^68^Ga]Ga-PSMA-11 uptake in total tumor load in patients with less than three metastases was expected to be minimal and thus the competition negligible. Patients were excluded if imaging followed previous treatment with [^177^Lu]Lu-PSMA-617, [^223^Ra]Ra-dichloride, or external beam radiation of the head/neck region. If a patient had more than one [^68^Ga]Ga-PSMA-11 PET/CT, the most recent one was included.

### 2.2. Preparation of Radioligand

[^68^Ga]Ga-PSMA-11 was prepared using a GMP-grade ^68^Ge/^68^Ga generator and a semi-automated Modular-Lab eazy synthesis module (Eckert & Ziegler, Berlin, Germany). Each synthesis was performed following the manufacturers’ instructions using prefabricated materials including a cassette, an acetate buffer, a C18 purification cartridge, and a 0.22-μm pore size sterilization filter (Eckert & Ziegler, Berlin, Germany). Forty micrograms (42 nmol) of PSMA-11 ligand (ABX, Radeberg, Germany) per preparation was used, leading to the mean administered amount of 14.5 (range 5.1–41.1) μg ligand per patient depending on patient weight and ^68^Ga decay.

### 2.3. Image Acquisition and Reconstruction

Images were acquired from the skull vertex to the mid-thigh using a Biograph mCT scanner (Siemens, Erlangen, Germany). Intravenous 1.5–2.0 MBq/kg [^68^Ga]Ga-PSMA-11 was administered followed by 500 mL of saline. The PET images were acquired 60 min after injection and after voiding. Low dose CT was done directly after PET. The PET reconstruction was according to EARL recommendations, even though its use for [^68^Ga]Ga-PSMA-11 PET/CT interpretation has not been validated yet [17].

### 2.4. Imaging Analysis

To quantify in vivo distribution of [^68^Ga]Ga-PSMA-11 in metastatic prostate cancer patients, image analysis was performed using the Syngo.via-software (Siemens version 05.01, Erlangen, Germany). Relevant volumes of interest were (semi)automatically segmented if the standardized uptake value peak (SUV_peak_) was greater than the threshold set by a 3-cm cylindrical region of interest (ROI) in the aorta adapted from the PERCIST criteria (threshold: 1.5 × aorta peak + 2 × standard deviation) [18]. The SUV_peak_ was corrected for lean body mass (SUV_lbm,peak_) according to the formula as defined in the EANM guidelines [19]. If individual organs were not automatically divided based on set PERCIST criteria (e.g., liver and right kidney), manual adoption was needed. Blood pool activity has shown to be a reliable reference region for [^68^Ga]Ga-PSMA-11 imaging interpretation [20]. Segmentations smaller than 0.3 mL were discharged. If there were more than 50 lesions segmented, the Syngo.via software automatically calculated the total amount of [^68^Ga]Ga-PSMA-11 uptake of the remaining lesions (>50) and did not visualize them on the [^68^Ga]Ga-PSMA-11 PET/CT.

Parameters collected were the PSMA-derived total tumor volume (TV-PSMA) in cm^3^, fractional healthy tissue [^68^Ga]Ga-PSMA-11 uptake volume (HT-PSMA) of the lacrimal, submandibular, and parotid glands, and kidneys (of all tissues the left and right separately), and the fractional total lesion [^68^Ga]Ga-PSMA-11 uptake volume (TL-PSMA) of the prostate cancer (summation of the entire tumor load within the patient). Both HT-PSMA and TL-PSMA were calculated according to the EARL recommendations by multiplying SUV_peak_ with the PSMA-derived tumor volume or in case of HT-PSMA, healthy tissue volume (SUV_lbm,peak_*cm^3^).

### 2.5. Outcome Measurement and Statistical Analysis

IBM SPSS Statistics version 25.0.0.2 for Windows (IBM, Armonk, NY, USA) was used for all the data analyses. The Shapiro–Wilk test was performed to test for normal distribution, with *p* < 0.05 deemed as significant.

For the correlation analyses, a bivariate correlation with a two-tail test for significance was used (Pearson if normally distributed and Spearman if not normally distributed), and *p* < 0.01 was deemed significant. A correlation coefficient between 0.90–1.00 was considered very strong; 0.70–0.89, strong; 0.40–0.69, moderate; 0.10–0.39, weak; and 0.00–0.10, negligible [21].

If a patient had a history of (partial) nephrectomy, the patient’s HT-PSMA of the kidneys was not included in the correlation analysis. If the lacrimal, submandibular, and parotid glands were not automatically segmented based on the threshold set by the blood pool activity, the value was set to zero for the analysis.

## 3. Results

**Figure 1:**
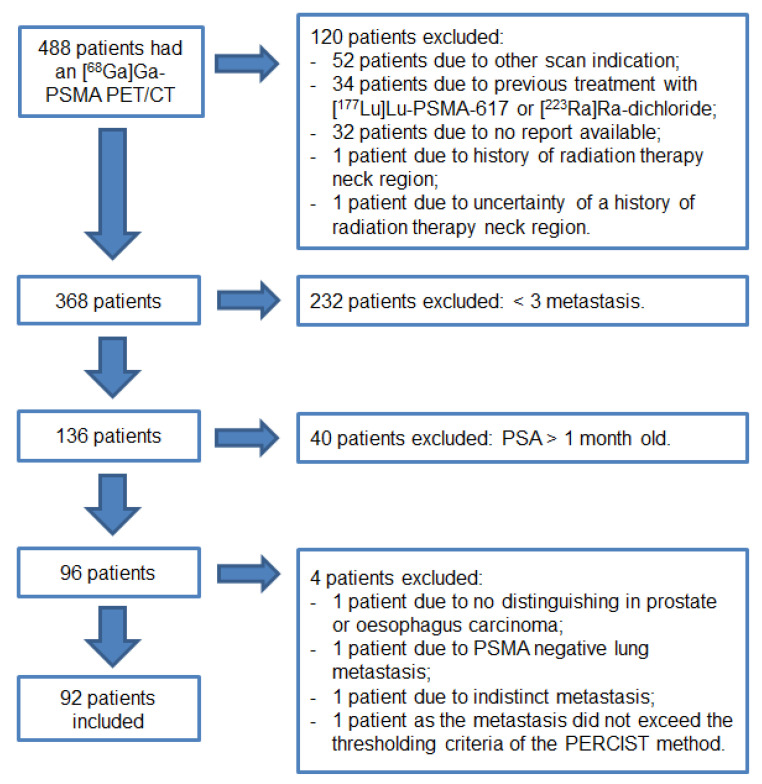
Flowchart of this retrospective study.

A total of 488 patients had a [^68^Ga]Ga-PSMA-11 PET/CT between March 2016 and January 2019. Ninety-two patients met the inclusion criteria as shown in Figure 1.

Six patients were imaged for primary staging of prostate cancer, 17 patients for biochemical recurrence, 36 patients for (biochemical) progression, and 33 patients were evaluated prior to [^177^Lu]Lu-PSMA-617 therapy or [^223^Ra]Ra-dichloride therapy. Baseline characteristics are summarized in Table 1.

Median PSA value was 51.50 ng/mL (IQR 5.50–167.50). Median TV-PSMA was 185.35 cm^3^ (IQR 32.76–555.12), median TL-PSMA was 703.37 SUV*cm^3^ (IQR 119.56–2778.20), median HT-PSMA of the lacrimal, submandibular, and parotid glands, and kidneys was 13.69 (IQR 7.29–19.06), 194.75 (IQR 133.67–276.53), 552.54 (IQR 379.98–737.16), and 8092.75 SUV*cm^3^ (IQR 5793.02–11,385.86), respectively. Median HT-PSMA of the salivary and the lacrimal glands combined was 780.97 SUV*cm^3^ (IQR 546.22–980.03).

**Figure 2:**
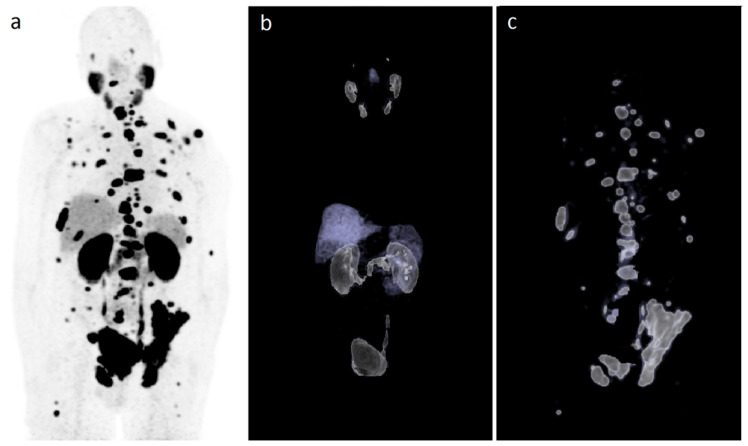
Illustration of the selected sites by the Syngo.via-software. (**a**) [^68^Ga]Ga-PSMA-11 PET/CT maximum intensity projection (MIP) of a patient with metastatic prostate cancer; (**b**) volume rendering technique (VRT) of the same patient’s [^68^Ga]Ga-PSMA-11 PET/CT showing only the healthy tissue PSMA uptake; (**c**) VRT of the same patient’s [^68^Ga]Ga-PSMA-11 PET/CT with the selected tumor sites by the Syngo.via-software including lymph nodes, bone, and visceral lesions, after manual deletion of healthy tissues PSMA uptake as shown in (**b**).

Figure 2 illustrates a case with selected sites by the Syngo.via-software for the analysis of TL-PSMA.

### Correlation Analysis

In all 92 patients, the submandibular- and parotid glands exceeded the threshold set by the blood pool activity. In four out of 92 patients (4%) the lacrimal gland(s) did not exceed the threshold set by the blood pool activity and was therefore set to zero. Six out of 92 patients (7%) had a history of (partial) nephrectomy; thus, these patients were excluded from the correlation analysis of the HT-PSMA of the kidneys.

**Figure 3:**
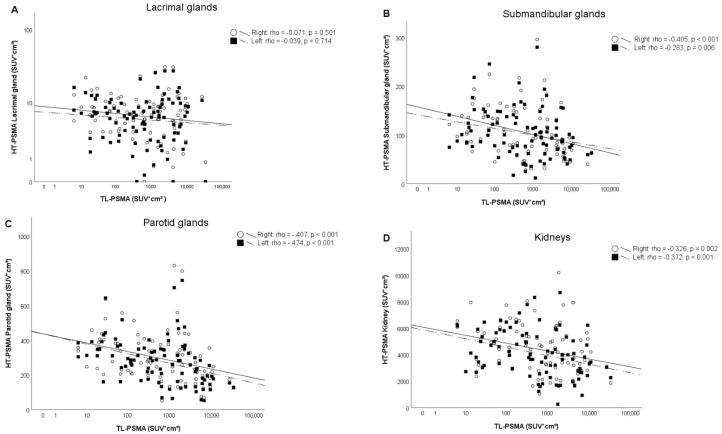
Correlation between the fractional uptake volume of [^68^Ga]Ga-PSMA-11 in different healthy tissues (HT-PSMA) divided by side and the fractional total lesion [^68^Ga]Ga-PSMA-11 uptake volume (TL-PSMA) in patients with metastatic prostate cancer. (**A**) Lacrimal glands; (**B**) submandibular glands; (**C**) parotid glands; and (**D**) kidneys. No correlation was found between the lacrimal glands and TL-PSMA. A weak–moderate correlation was found between the TL-PSMA and the submandibular- and parotid glands and kidneys, with a correlation coefficient of −0.447, −0.345, and −0.394, respectively (when left and right side are combined). Rounds/continuous line represents the right side of the organ, squares/dashed line represents the left side of the organ, with the correlation coefficient of the separate sides presented in the upper right corner of each graph.

A significant (Spearman, *p*-value ≤ 0.001) but weak–moderate correlation was found between the TL-PSMA and HT-PSMA of the submandibular- and parotid glands and kidneys (left and right side combined) with a correlation coefficient of −0.345, −0.447, and −0.394, respectively (Figure 3). There was also a significant (Spearman, *p*-value < 0.001) but moderate correlation found between the TL-PSMA and HT-PSMA of the glands combined with a correlation coefficient of −0.459.

In addition, a significant (Spearman, *p*-value ≤ 0.001) but weak–moderate correlation was found between the TV-PSMA and HT-PSMA of the submandibular- and parotid glands and kidneys (left and right side combined) with a correlation coefficient of –0.339, −0.482, and −0.374, respectively.

**Figure 4:**
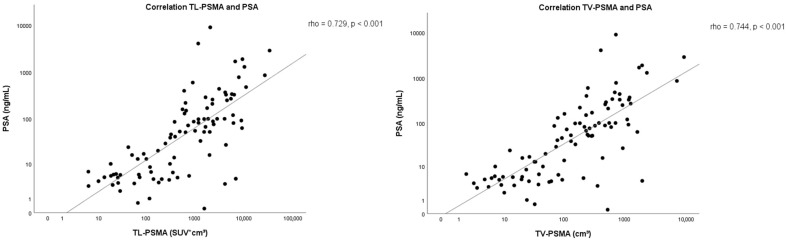
Correlation between the PSA value and the fractional total lesion [68Ga]Ga-PSMA-11 uptake volume (TL-PSMA) and PSA and the PSMA-derived total tumor volume (TV-PSMA) in men with metastatic prostate cancer.

**Figure 5:**
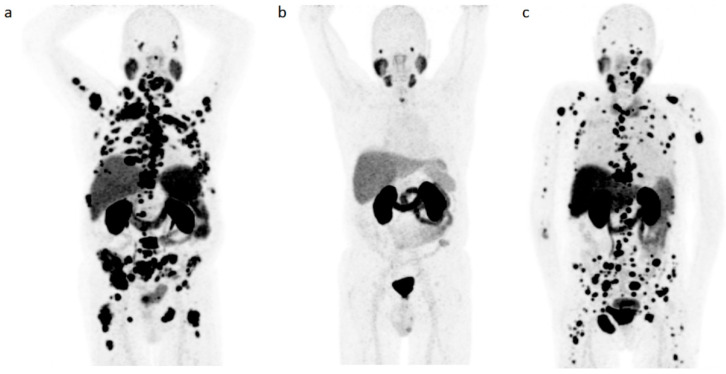
Example of [^68^Ga]Ga-PSMA-11 PET/CT maximum intensity projection (MIP) of three patients with prostate cancer and different fractional total lesion [^68^Ga]Ga-PSMA-11 uptake volume (TL-PSMA) with almost similar (**a**,**b**) and different (**a**–**c**) fractional [^68^Ga]Ga-PSMA-11 uptake volume in the parotid glands (HT-PSMA parotid glands). (**a**) PSA: 63 ng/mL, administered ^68^Ga-PSMA-11: 1.59 Mbq/kg, TL-PSMA: 9305.29 SUV*cm^3^, HT-PSMA parotid glands: 327.69 SUV*cm^3^; (**b**) PSA: 4.60 ng/mL, administered ^68^Ga-PSMA-11: 1.54 Mbq/kg, TL-PSMA: 26.00 SUV*cm^3^, HT-PSMA parotid glands: 363.36 SUV*cm^3^; (**c**) PSA: 340 ng/mL, administered ^68^Ga-PSMA-11: 1.61 Mbq/kg, TL-PSMA: 5767.73 SUV*cm^3^, HT-PSMA parotid glands: 133.23 SUV*cm^3^.

A significant and strong correlation (Spearman, *p* < 0.001) was found between TL-PSMA and PSA value with a correlation coefficient of 0.729 and TV-PSMA and PSA with a correlation coefficient of 0.744 (Figure 4). No correlation was found between TL-PSMA and HT-PSMA of the lacrimal glands (Figure 3) and the TV-PSMA and HT-PSMA of the lacrimal glands. An overview of the results is shown in Table 2. An example of similar and different HT-PSMA in different TL-PSMA is depicted in Figure 5.

## 4. Discussion

In the 92 men evaluated, the influence of TL-PSMA on [^68^Ga]Ga-PSMA-11 uptake in different healthy tissues was limited and only weak–moderate correlated for the salivary glands and kidneys. As a secondary outcome, a clear correlation between PSA value and TL-PSMA was found. Similar results were found with additional tests with TV-PSMA alone; however, this is expected due to collinearity with TL-PSMA (as TL-PSMA = fractional uptake (in SUV_lbm,peak_) * TV-PSMA (in cm^3^)).

These results support the findings of Gaertner et al. and Filss et al., who also found a correlation between [^68^Ga]Ga-PSMA-11 uptake in salivary glands and kidneys, and tumor uptake [14,15], suggesting that a ‘steal’ phenomenon exists. However, in the current study no correlation was found between [^68^Ga]Ga-PSMA-11 uptake in the lacrimal glands and TL-PSMA, which is in contrast with the findings of Gaertner et al., who did find a correlation [15].

As a limitation, Gaertner et al. divided tumor uptake in three categories (low, medium, and high) based on the visual assessment of tumor load on the on [^68^Ga]Ga-PSMA-11 PET/CT, which makes it hard to directly compare these findings with our results [15]. Although it is easier to apply visual classifications in practice, the definition of low and medium tumor load in their study was subjective and visual interpretations are known to be prone to inter-observer variability. Another reason why they apply visual interpretations may be because no validated quantitative measurements for [^68^Ga]Ga-PSMA-11 PET/CT reconstructions are currently available. The use of EARL recommendations for HT-PSMA and TL-PSMA in this study was chosen because, even though these recommendations are used for [^18^F]-FDG (FDG), the used method for the total lesion glycolysis (TLG) will give the best impression of in vivo distribution of [^68^Ga]Ga-PSMA-11 by calculating the fractional tumor activity [16,22,23].

Additionally, Filss et al. had a small sample size of only eleven patients and did not evaluate the [^68^Ga]Ga-PSMA-11 uptake in the lacrimal glands [14]. Nonetheless, the correlations found by Gaertner et al. and in the current study between TL-PSMA and [^68^Ga]Ga-PSMA-11 uptake in healthy tissues were weak–moderate at best [15].

Earlier dosimetry studies on Luthetium-177 labeled PSMA-targeted radioligand therapy also demonstrated physiological uptake by the lacrimal glands, salivary glands (parotid glands and submandibular glands) and the kidneys and thereby exposed to radiation [24,25,26]. To the best of our knowledge, the study by Violet et al. is the only study to evaluate the correlation between tumor volume and the absorbed [177Lu]Lu-PSMA-617 dose in healthy tissue. In their population of 30 patients treated with [177Lu]Lu-PSMA-617, an inverse correlation between tumor volume (defined on the PSMA PET/CT) and the mean dose to the parotids glands and kidneys was found with a correlation coefficient of −0.41 (*p* = 0.03) and −0.43 (*p* = 0.02), respectively [27]. Those results are in line with our findings.

The weak–moderate correlation on TL-PSMA and [^68^Ga]Ga-PSMA-11 uptake in salivary glands and kidneys in this study suggests that the healthy tissue uptake of [^68^Ga]Ga-PSMA-11 is only partially influenced by TL-PSMA.

Literature on PSMA expression in healthy tissues is contradictory. Some describe physiological PSMA expression on the renal tubular cells and salivary glands in a much lower level than prostate cancer tissue [28,29], while other publications report no PSMA expression in salivary glands [30,31,32]. Rupp et al. found that there was mainly a non-PSMA-related radioligand uptake in the salivary glands [33], which supports the hypothesis that other factors are involved. In vitro studies have shown that androgen deprivation therapy (ADT) increases the PSMA expression in prostate cancer cells [34,35]. This was later confirmed in clinical studies, showing increased PSMA expression in prostate tumor tissue as well as in healthy tissue, especially in the salivary glands, after ADT initiation [36,37]. It is not fully understood yet why this is most notable in the salivary glands; however, it seems that the intercellular blockage of androgen synthesis not only increases the expression of the androgen receptor but also the expression of PSMA.

This further supports the weak–moderate correlation between the TL-PSMA and HT-PSMA (salivary glands and kidneys) found in our study and suggests that healthy tissue uptake of [^68^Ga]Ga-PSMA-11 is not solely influenced by TL-PSMA.

In contrast, a strong correlation was found between PSA value and the TL-PSMA of the prostate cancer. This is in line with previous research by Brito et al. and Schmidkonz et al., suggesting that PSA values can be used as a biomarker for tumor uptake [22,38]. Unfortunately, in the study of Brito et al. and Schmidkonz et al. it was not clear how old the PSA values were. Their results could therefore be less accurate [22,38].

This study had several limitations. First of all, the choice to only include patients with three or more metastasis was arbitrary but chosen under the assumption that difference in ligand binding is only expected in case of sufficient competition between tumor and healthy tissue. Secondly, the Syngo.via-software could not always automatically divide different tissues (e.g., liver and right kidney) based on a PERCIST-based blood pool activity threshold method. Therefore, manual adaption was needed, which could give inter-observer variability. In addition, if there were more than 50 lesions segmented based on blood pool activity, the remaining lesions (exceeding 50) were not displayed by the software and could not be visually verified as tumor tissue, and had to be assumed as tumor tissue. This might have resulted in a slight overestimation of TL-PSMA in patients with an already high tumor load.

Thirdly, the included patients belong to a heterogeneous population with different previous treatments which may have an influence on our results. Some patients were referred from another center and therefore crucial information on treatment history and medication (i.e., ADT) was not always available. However, despite of the heterogeneous patient population a significant weak–moderate correlation was still found between TL-PSMA and HT-PSMA, suggestive that a ’steal’ phenomenon exists but cannot be confirmed.

## 5. Conclusions

The existence of a ‘steal’ phenomenon cannot be confirmed in this study. Healthy tissue uptake of [^68^Ga]Ga-PSMA-11 is only partially influenced by TL-PSMA. Thus, modification of therapeutic PSMA activity should not be adjusted based on TL-PSMA alone. Further studies will be necessary to investigate the exact mechanism of [^68^Ga]Ga-PSMA-11 uptake in different healthy tissues to identify the different factors involved.

## Figures and Tables

**Table 1:** Baseline characteristics.

Characteristic	Result	*p*-Value
Total patients, *n*	92	
Indication for [^68^Ga]Ga-PSMA-11 PET/CT, *n* (%):		
• Primary staging	6 (7%)	-
• Biochemical recurrence	17 (18%)	-
• (Biochemical) progression	36 (39%)	-
• Evaluated prior to radioligand therapy	33 (36%)	-
Age, years (mean, SD)	71 (8)	0.481
Weight, kg (median, IQR)	80 (74–90)	0.001 *
Administered [^68^Ga]Ga-PSMA-11, MBq/kg (median, IQR)	1.58 (1.51–1.65)	0.000 *
Incubation time, minutes (mean, SD)	62 (11)	0.119
PSA value, ng/mL (median, IQR)	51.50 (5.50–167.50)	0.000 *
TV-PSMA, cm^3^ (median, IQR)	185.35 (32.76–555.12)	0.000 *
TL-PSMA, SUV*cm^3^ (median, IQR)	703.37 (119.56–2778.20)	0.000 *
HT-PSMA, SUV*cm^3^ (median, IQR) ^1^ (mean, SD):		
• Lacrimal glands:	13.69 (7.29–19.06)	0.000 *
left	6.50 (3.17–9.74)	0.000 *
right	7.05 (3.72–10.44)	0.000 *
• Submandibular glands:	194.75 (133.67–276.53)	0.003 *
left	97.04 (70.33–137.16)	0.005 *
right	95.56 (67.76–138.38)	0.004 *
• Parotid glands:	552.54 (379.98–737.16)	0.000 *
left	282.79 (176.16–350.36)	0.001 *
right	272.30 (201.63–376.21)	0.000 *
• Combined glands **	780.97 (546.22–980.03)	0.000 *
• Kidneys:	8092.75 (5793.02–11,385.86)	0.014 *
left ^1^	4113.43 (1747.59)	0.442
right ^1^	4420.92 (1802.97)	0.073

Legend: HT-PSMA = fractional healthy tissue [^68^Ga]Ga-PSMA-11 uptake volume, IQR = interquartile range, SD = standard deviation, TL-PSMA = fractional total lesion [^68^Ga]Ga-PSMA-11 uptake volume, TV-PSMA = PSMA-derived total tumor volume, PSA = prostate-specific antigen. * Significant *p*-values Shapiro–Wilk (*p* < 0.05). ** Combined glands include: the salivary glands and the lacrimal glands.

**Table 2:** Correlation outcomes of HT-PSMA and PSA.

**Parameters**	**Total Patients**	**TL-PSMA**		**TV-PSMA**	
	*n*	*p*-value	rho	*p*-value	rho
HT-PSMA:					
- Left lacrimal gland	92	0.714	−0.039	0.567	−0.061
- Right lacrimal gland	92	0.501	−0.071	0.419	−0.085
- Total lacrimal glands	92	0.599	−0.056	0.491	−0.073
- Left submandibular gland	92	0.006 *	−0.283	0.001 *	−0.345
- Right submandibular gland	92	0.000 *	−0.405	0.000 *	−0.447
- Total submandibular glands	92	0.001 *	−0.345	0.000 *	−0.399
- Left parotid gland	92	0.000 *	−0.474	0.000 *	−0.502
- Right parotid gland	92	0.000 *	−0.407	0.000 *	−0.451
- Total parotid glands	92	0.000 *	−0.447	0.000 *	−0.482
- Combined glands **	92	0.000 *	−0.459	0.000 *	−0.500
- Left kidney	88	0.000 *	−0.372	0.001 *	−0.359
- Right kidney	90	0.002 *	−0.326	0.002 *	−0.322
- Total kidneys	86	0.000 *	−0.394	0.000 *	−0.374
PSA-level	92	0.000 *	0.729	0.000 *	0.744

Legend: HT-PSMA = fractional healthy tissue [^68^Ga]Ga-PSMA-11 uptake volume (cm^3^*SUV), TL-PSMA = fractional total lesion [^68^Ga]Ga-PSMA-11 uptake volume (cm^3^*SUV), TV-PSMA = PSMA-derived total tumor volume (cm^3^), PSA = prostate specific antigen (ng/mL). The Spearman rank test was used for all the correlation analyses. * Significant *p*-values (<0.01). ** Combined glands include: the salivary glands and the lacrimal glands.

## Data Availability

The data presented in this study are available on request from the corresponding author. The data are not publicly available due to privacy regulations.
